# Refining MDR-TB treatment regimens for ultra short therapy (TB-TRUST): study protocol for a randomized controlled trial

**DOI:** 10.1186/s12879-021-05870-w

**Published:** 2021-02-17

**Authors:** Taoping Weng, Feng Sun, Yang Li, Jiazhen Chen, Xinchang Chen, Rong Li, Shijia Ge, Yanlin Zhao, Wenhong Zhang

**Affiliations:** 1 Departments of Infectious Diseases, Huashan Hospital, Fudan University, Shanghai, 200040 China; 2 National Center for Tuberculosis Control and Prevention, Chinese Center for Disease Control and Prevention, Beijing, China

**Keywords:** Tuberculosis, Multidrug-resistant tuberculosis, Multicenter, Randomized trial, Non-inferiority, Precision treatments, Ultra-short regimen

## Abstract

**Background:**

Multidrug-resistant tuberculosis (MDR-TB) are unsatisfied to treat, pressing more effective and innovative treatment regimens. New efficient regimens for MDR-TB have obtained high treatment success rates. However, those regimens without drug susceptibility testing (DST) are also likely to contribute to the emergence of resistance. Precision treatments guided by DST might optimize the patients’ treatment outcome individually and minimize resistance amplification.

**Methods:**

TB-TRUST is a phase III, multicenter, open-label, randomized controlled clinical trial of non-inferiority comparing the treatment success rate between the World Health Organization (WHO) shorter regimen and the refined ultra-short regimen for fluoroquinolones and second-line injectable drugs susceptible rifampicin-resistant TB. The control arm uses the WHO injectable-containing shorter regimen for 36–44 weeks depending on time of sputum smear conversion. The investigational arm uses a refined ultra-short regimen guided by molecular DST to pyrazinamide via whole-genome sequencing (WGS) to optimize the treatment of pyrazinamide-susceptible patients with levofloxacin, linezolid, cycloserine and pyrazinamide for 24–32 weeks and pyrazinamide-resistant with levofloxacin, linezolid, cycloserine and clofazimine for 36–44 weeks. The primary outcome is the treatment success rate without relapse at 84 weeks after treatment initiation. Secondary outcomes include the time of sputum culture conversion and occurrence of adverse events. Assuming α = 0.025 level of significance (one-sided test), a power of 80%, a < 10% difference in treatment success rate between control arm and investigational (80% vs. 82%), and a 5% lost follow-up rate, the number of participants per arm to show non-inferiority was calculated as 177(354 in total).

**Discussion:**

Rapid molecular testing distinguishes patients who are eligible for shorter regimen with fluoroquinolone and the WGS-guided results shorten the treatment to 6 months for pyrazinamide susceptible patients. It’s foreseeable that not only novel developed medicines, but also traditional powerful medicines with the susceptibility confirmed by DST are the key factors to ensure the effect of anti-MDR-TB drugs. As a DST-guided precision treatment, TB-TRUST are expected to optimize therapy outcome in more patients who cannot afford the expensive new medicines and minimize and even avoid resistance amplification with the rational use of anti-TB drugs.

**Trail registration:**

ClinicalTrial.gov, NCT03867136. Registered on March 7, 2019.

**Supplementary Information:**

The online version contains supplementary material available at 10.1186/s12879-021-05870-w.

## Background

As an ancient infectious disease, tuberculosis (TB) has gained new worldwide attention due to the emergence of drug resistance. About 20% of the TB strains have developed resistance to at least one anti-TB drug. In 2019, there were approximately 465,000 incident cases of rifampicin-resistant TB (RR-TB), of which 78% had multidrug-resistant TB (MDR-TB) [1].

MDR-TB is heavily challenging to treat, pressing more powerful and novel treatment regimens. Treatment for MDR-TB is largely carried out through standardized, empirical combination regimens. However, precision treatment guided by detailed DST results in improved individual outcomes [2]. Standardized regimens are expected to improve access to treatment, but they are also likely to contribute to the emergence of resistance. On the contrary, a DST-guided precision medicine will improve therapy outcome and minimize resistance amplification at the same time.

### Study of short regimen for MDR-TB treatment

Treatment of MDR-TB remains challenging, with lengthy treatment durations and complex drug regimens. In 2010, Van et al. reported he Bangladesh regimen of 9-month consisting of seven drugs for MDR-TB treatment, resulting in a treatment success rate of 87.9%, marking the success of the short-term treatment for MDR-TB [3]. Subsequently, similar confirmatory studies were carried out in Cameroon [4] and Niger [5], all of which obtained extremely high treatment success rates over 89%. In 2019, A.J. Nunn et al. published the first randomized controlled trial (RCT) study on short-term treatment of MDR-TB. The standardized shorter regimen of 9–11 months composed of 7 drugs with a treatment success rate of 78.8% was proved to be non-inferiority with the long-term program recommended by the World Health Organization (WHO) in 2011 [6]. After reviewing the results of the STREAM study and other observational studies, WHO released updated guidelines for MDR-TB in 2018 that introduced shorter regimen as an option for patients who have not been previously treated for more than 1 month with second-line medicines or have no evidence of resistance to fluoroquinolones and second-line injectable drugs (SLIDs) [7]. In 2020, Conradie F et al. published the outcome of Nix TB study [8]. They investigated the regimen of three oral drugs including bedaquiline, pretomanid and linezolid given for 26 weeks in patients with extensively drug-resistant (XDR) TB. Totally, 109 patients were enrolled in the study and at the end of treatment, 98 patients (90%) had a favorable outcome, which suggested the combination of bedaquiline, pretomanid, and linezolid leading to a favorable outcome among a high percentage of patients with fluoroquinolones resistance.

In our early study, we showed that introducing molecular DST to pyrazinamide successfully improved the treatment outcome with the relapse-free success rate of 82.4% for pyrazinamide-susceptible cases and shortened the regimen to 12 months without any additional agents [9]. However, concerning its limits of nonrandomized design and relatively small sample sizes, we here launched this randomized controlled trial, Refining MDR-TB Treatment Regimens for Ultra Short Therapy (TB-TRUST), with the objective to evaluate the effectiveness and feasibility of a novel all-oral ultra-short regimen with the guidance of molecular DST to pyrazinamide.

### Innovative DST technology for fluoroquinolones, SLIDs and pyrazinamide

Rapid testing of resistance to fluoroquinolones and SLIDs is of great significance for recognizing MDR-TB patients who are eligible and recommended in shorter regimens. Xpert® MTB/XDR (Cepheid, Sunnyvale, CA, USA) leverages the new 10-colour homogeneous detection within the GeneXpert system, which enables the detection of multiple mutations across several genes with detecting resistance to isoniazid, ethionamide, fluoroquinolones, amikacin, kanamycin and capreomycin. Xpert® MTB/XDR testing can quickly select fluoroquinolones and SLIDs susceptible patients taking the most appropriate and precise shorter regimen [10].

Pyrazinamide resistance is associated with full-length mutations in *pncA* [11, 12], which can mainly detected through culturing strains of *Mycobacterium tuberculosis* (MTB). Whole-genome sequencing (WGS) is a viable and financially feasible tool for timely and comprehensive diagnosis of drug resistance, and our early study has proved it a promising approach to predict resistance to pyrazinamide with satisfactory accuracy, sensitivity and specificity of over 85.0% [13]. Our central laboratory, which has been studying resistant mechanism of MTB for a long time and has discovered many resistance-related mutant sites [14–17], is able to interpret the result of the WGS to detect pyrazinamide resistance and review fluoroquinolones and SLIDs resistance tested by Xpert® MTB/XDR, which makes precising and optimizing MDR-TB treatment regimen possible.

### Advanced RCT study with DST for pyrazinamide

TB-TRUST is designed based on the trend of shorter and full oral treatment programs under DST guidance. Based on the molecular susceptibility testing platforms of Xpert® MTB/XDR and WGS, a randomized controlled study is carried out. This study innovatively detects fluoroquinolone and SLIDs resistance to select opportune rifampicin-resistant/multidrug-resistant tuberculosis (RR/MDR-TB) patients and optimizes the regimen according to result of pyrazinamide resistance, comparing with the WHO shorter regimen as a standardized and non-DST guided control arm. This protocol describes the background, design and rationale of the TB-TRUST trial.

## Methods

### Study regimen and comparator

**Fig. 1:**
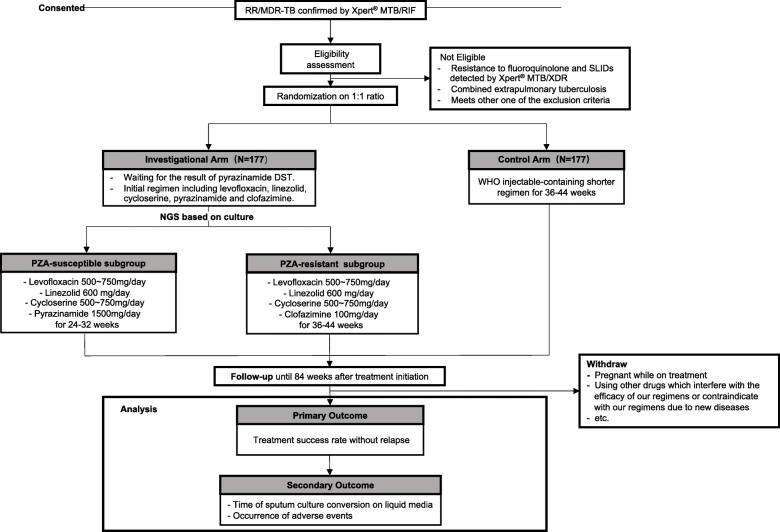
TB-TRUST study schematic. The study plan to recruit RR/MDR-TB patients who are susceptible to fluoroquinolones and second-line injectable agents. Recruited patients will be randomly divided into the ultra-short regimen group and the WHO shorter treatment with a ratio of 1:1. For patients in the ultra-short regimen, DST of WGS to pyrazinamide will be performed via using the culture isolates. During the first 4–8 weeks, patients are waiting for the result of pyrazinamide DST and receive five agents including levofloxacin, linezolid, cycloserine, pyrazinamide and clofazimine. After molecular pyrazinamide DST results are obtained, pyrazinamide-susceptible patients for 24–32 weeks depending on time to sputum smear conversion receiving agents including levofloxacin, linezolid, cycloserine, pyrazinamide, while pyrazinamide-resistant for 36–44 weeks receiving agents including levofloxacin, linezolid, cycloserine, clofazimine. The control arm uses the WHO injectable-containing shorter regimen for 36–44 weeks depending on time to sputum smear conversion. Follow-up visits were conducted every 2 weeks in the first 8 weeks after treatment initiation, and then followed every 4 weeks until the end of treatment, and every 12 weeks after discontinuation until 84 weeks.(Abbreviations in this figure: RR/MDR-TB: rifampicin-resistant/multidrug-resistant tuberculosis. SLID: second-line injectable drugs. NGS: next generation sequencing. PZA: pyrazinamide)

This is a phase III, multicenter, open-label, randomized controlled clinical trial of non-inferiority with two arms, recruiting RR/MDR-TB patients susceptible to fluoroquinolones and SLIDs detected by Xpert® MTB/XDR. Recruited participants will be randomly divided into the ultra-short regimen as investigational arm and the WHO shorter regimen as control with a ratio of 1:1. Figure 1 shows the flow chart of TB-TRUST trail.

The ultra-short regimen consists of two periods. During the first 4–8 weeks, patients are waiting for the result of pyrazinamide DST and receive five agents including levofloxacin, linezolid, cycloserine, pyrazinamide and clofazimine. The result of molecular DST to pyrazinamide will be determined by two technical staff in the central laboratory of Huashan Hospital, Fudan University according to the established criteria that were reported previously in elsewhere [18, 19], refining the ultra-short regimen. After WGS results including pyrazinamide resistance are obtained, patients will be divided into two subgroups: pyrazinamide-susceptible patients and pyrazinamide-resistant patients. For pyrazinamide-susceptible patients, clofazimine will be discontinued while levofloxacin, linezolid, cycloserine and pyrazinamide are given until 24th week (prolonged to 28 or 32 weeks if smear has not converted by 16 or 20 weeks). The regimen for pyrazinamide-resistant patients consists of levofloxacin, linezolid, cycloserine and clofazimine given until 36th week (prolonged to 40 or 44 weeks if smear has not converted by 16 or 20 weeks). If the sputum culture is always negative resulting in the unavailability of WGS, the treatment regimen of the five drugs will be continued to 36 weeks (prolonged to 40 or 44 weeks if smear has not converted by 16 or 20 weeks).

The control regimen is WHO injectable-containing shorter regimen with two phases of treatment for 36–44 weeks. The first is the intensive phase of 16 weeks (extended up a maximum of 20 or 24 weeks in case of lack of smear conversion by 16 or 20 weeks), containing moxifloxacin, amikacin, prothionamide, pyrazinamide, high-dose isoniazid, ethambutol and clofazimine. The followed one is a continuation phase for 20 weeks with the following agents: moxifloxacin, pyrazinamide, ethambutol and clofazimine. Table 1 shows the doses and usage of each drug in the WHO shorter regimen and the ultra-short regimen.

**Table 1:** TB-TRUST study regimen drugs and doses

Drug	Weight group		Usage
	≤50 kg	> 50 kg	
Pyrazinamide	1500 mg daily		Once, before or after meal
Levofloxacin	500 mg daily	750 mg daily	Once, before or after meal
Linezolid	600 mg daily		Once, before or after meal
Cycloserine	500 mg daily	750 mg daily	Once or divide in two, before or after meal
Clofazimine	100 mg daily		Once, before or after meal
Moxifloxacin	400 mg daily		Once, before or after meal
Prothionamide	500 mg daily	750 mg daily	Once, before or after meal
Ethambutol	750 mg daily	1000 mg daily	Once, before or after meal
Amikacin	600 mg daily		Once, intramuscularly
Isoniazid	600 mg daily		Once, before or after meal

### Site selection

This trail is under the guidance of the China National Tuberculosis Defense Association, led by Huashan Hospital, Fudan University, Zhejiang Province Center for Disease Control and Prevention and Chinese Center for Disease Control and Prevention. The recruiting-cooperative units are 15 hospitals in nine provinces in China, namely Henan Infectious Disease Hospital, Xinjiang Uygur Autonomous Region Chest Hospital, Shenzhen Third People’s Hospital, Hangzhou Red Cross Hospital, Ningbo Huamei Hospital of Chinese Academy of Sciences (Ningbo Second Hospital), Enze Hospital of Enze Medical Center (Group) in Taizhou, Zhejiang, the Affiliated Hospital of Shaoxing College of Arts and Sciences (Shaoxing Municipal Hospital), Guiyang Public Health Treatment Center, Wenzhou Central Hospital, Jiangxi Chest Hospital, Hunan Chest Hospital, Huaihua First People’s Hospital, Shanxi Tuberculosis Prevention and Treatment Institute, Xuzhou Infection Disease Hospital, Yunan Infectious Diseases Hospital. A specialized auditing team will make the quality control of the cooperative units half a year.

### Patient eligibility criteria

In addition to participate in this clinical trial voluntarily, the main eligibility criterion is smear-positive pulmonary RR/MDR-TB susceptible to fluoroquinolones and SLIDs identified by the Xpert® MTB/XDR platform. Patients will be excluded if they were in critical condition or with aspartate transaminase or alanine aminotransferase greater than five times the upper limit of normal, or blood creatinine greater than 1.5 times the upper limit of normal or HIV testing positive, considering the safety in the regimen. If patients are simultaneously applying the drugs that affect the efficacy of or contraindicated with this study drugs, they will be also excluded. Besides, pregnant or breastfeeding women are also excluded. Table 2 shows the detailed inclusion and exclusion criteria. Those patients are pregnant while on treatment and using other drugs which interfere with the efficacy of our regimens or contraindicate with our regimens due will withdraw, who will then be treated by Chinese national guideline for RR/MDR-TB.

**Table 2:** TB-TRUST inclusion and exclusion criteria

	Detailed Description
Inclusion criteria	1) Willing to participate in trial treatment, follow-up and sign informed consent 2) Age 18–70 years old 3) Smear-positive pulmonary tuberculosis with resistance to rifampicin confirmed by Xpert® MTB/RIF (Cepheid, Sunnyvale, CA, USA) 4) Willing to carry out HIV testing 5) If a non-menopausal woman, willing to use or have used effective contraception during treatment 6) Have an identifiable address and stay in the area during the study period 7) Willing to follow the follow-up study procedure after the follow-up.
Exclusion criteria	1) Resistant to second-line injection confirmed by Xpert® MTB/XDR (Cepheid, Sunnyvale, CA, USA) 2) Resistant to fluoroquinolone confirmed by Xpert® MTB/XDR 3) Combined extrapulmonary tuberculosis 4) HIV antibody positive and AIDS patients 5) Severe patients and impossible to survive for more than 16 weeks according to the judgment of the research physician 6) Have been pregnant or breastfeeding 7) Unable to attend or follow treatment or follow-up time 8) Cannot take oral medications 9) Patients with impaired liver function (hepatic encephalopathy, ascites; total bilirubin is more than 2 times higher than the upper limit of normal; ALT or AST is more than 5 times the upper limit of normal) 10) Blood creatinine is more than 1.5 times the upper limit of normal 11) The investigator believes that there are any social or medical harms that expose to the participants 12) Simultaneously apply the drugs (glucocorticoids, interferons) that affect the efficacy of this study; and apply the following drugs contraindicated with the study regimens, including non-steroidal anti-inflammatory drugs, monoamine oxidase inhibitors (phenethyl hydrazine, different Carbofurs et al), direct or indirect sympathomimetic drugs (such as pseudoephedrine), vasopressor drugs (such as: adrenaline, norepinephrine), dopamine drugs (such as: dopamine, dobutamine), 5-HI reuptake inhibitor, a tricyclic antidepressant, a 5-HTI receptor antagonist (amitriptyline), meperidine or buspirone 13) Being allergic or intolerant of any study drug 14) Currently participating in another drug clinical trial 15) QTc interval ≥ 500 ms during screening 16) Hemoglobin is less than 90 g/L or platelet is less than 75*10^9/L 17) Have epilepsy, severe depression, irritability or psychosis 18) Alcohol abuse

### Recruitment process

RR/MDR-TB patients identified by Xpert® MTB/RIF assay (Cepheid, Sunnyvale, CA, USA) are screening to ascertain whether they satisfy the other eligibility criteria for enrolment. The follow-up content of this trial during the first screening including medical history collection, personal information collection, signed informed consent, physical examination, clinical evaluation, height and weight measuring, hearing examination, sputum smear, sputum culture, liver function, AIDS testing, blood sugar, blood potassium, electrocardiogram, chest radiography, urine routine, peripheral neuropathy brief examining with the use of a Brief Peripheral Neuropathy rating scale and ophthalmologic examination, including assessment of visual acuity and color vision. After screening procedures finished, potentially eligible patients are performed by Xpert® MTB/XDR to detect the resistance of fluoroquinolone and SLIDs, those without evidence of resistance to fluoroquinolone and SLIDs are recruited into this trial.

### Treatment allocation

The Patients who meet the eligibility criteria will be registered online and managed by an authorized doctor in every branch centers. And then participants will be assigned to the study arm based on an 1:1 randomization ratio. The randomization is performed through the online central randomization system stratified by the study sites (sponsored by Clinflash, China), and the treatment plan is notified to the branch centers by the central laboratory of Huashan Hospital, Fudan University.

### Duration of follow-up

After screening and baseline assessment, follow-up visits were conducted every 2 week in the first 8 weeks, and then every 4 weeks until the end of treatment, and every 12 weeks at the period of post-treatment until 84 weeks after treatment initiation. During the follow-up, physical examination, clinical assessment, weight measurement, sputum smear, sputum culture, blood and urine routine, liver function, renal function, electrocardiogram, brief peripheral neuropathy screen, ophthalmologic examination and some other tests will be evaluated. Detailed schedule of patient monitoring exams and laboratory tests will be shown in the additional file. To promote adherence to intervention, patients are asked to keep a drug-tablet taking diary and they will get allowance after completion of follow-up. Study enrollment began in June 2020 and is expected to finished in about half a years.

### Sample size assumptions

This sample size was calculated using the PASS 11 system (NCSS, Version: 11.0.10). Assuming α = 0.025 level of significance (one-sided test), a power of 80% and a non-inferiority margin of 10% between the control and investigational arms (80% versus 82% favorable outcome), the number of participants needed per arm to show non-inferiority of the investigational regimen was calculated as 168. Considering the 5% lost follow-up rate, 177 patients were required for each are, so the total sample size of the two arms was considered to be 354.

### Data collection and quality management

Clinic staff in the cooperative hospitals or centers will use the specialized website and data collection forms for study management to record data. All data access in websites will be controlled by unique usernames and passwords and staff will have access restricted to the functionality and data that are appropriate for their role in the study. Central Coordinating Office (CCO) staff will be responsible for provision of the relevant website and case report forms. CCO will check the eligibility of patients and follow-up data integrity regularly. After patients complete the trial, CCO will lock the file for further generation of data extracts for analysis. Only three main investigators of Huashan Hospital, Fudan University can have access to the final trial dataset and report the results of the trial via publication.

### Adverse event (AE) management

The grading system for AE has been established. To provide the advice to complicated event management, we build a highly experienced clinician team which includes a pulmonologist, an infectious diseases expert, a nephrologist, a psychiatrist, a cardiologist, a hepotologist, a hematologist, a rheumatologist, and an experienced nurse. Severe AE will be reported to the center within 24 h and discussed by the committee. AE and the adjustment or withdraw of drugs will be recorded and checked by CCO.

### External quality control of phenotypic DST

Baseline *M.tuberuclosis* strains from each patients enrolled in this study were sent to the National Tuberculosis Reference Laboratory of Chinese Center for Disease Control and Prevention in accordance with international transportation guidelines. A total of twelve anti-tuberculosis drugs susceptibility, including ofloxacin, moxifloxacin, rifampin, amikacin, rifabutin, para-aminosalicylic acid, ethionamide, isoniazid, kanamycin, ethambutol, streptomycin, and cycloserine were measured with the use of the Sensitire MYCOTB MIC Plate (MYCOTB) on one 96-wells plate.

### Assessment and analysis of outcome

The primary objective is to compare the treatment success rate without relapse between the WHO injectable-containing shorter regimen group and the refined ultra-short regimen group at 84 weeks after treatment initiation. Treatment outcomes will be classified into favorable outcome which means successful treatment and unfavorable outcome. Table 3 describes the definition of favorable and unfavorable outcome. Secondary outcomes including the time of sputum culture conversion and AEs are evaluated as well.

**Table 3:** Definition of the favorable and unfavorable outcomes in TB-TRUST study

Outcome	Description
Favorable outcome	• A participant’s outcome will be classified as favorable if their last two culture results are negative unless they have previously been classified as unfavorable. These two cultures must be taken on separate visits (on different days); the latest of which being within the Week 84 window (that is no more than 6 weeks before 84 weeks since randomization but with no upper bound). • Participants that don’t have a culture result within the Week 84 window because they were unable to produce sputum, will be classified as favorable if their last two cultures before the Week 84 window are negative and they have not previously been classified as unfavorable; such participants will be identified separately in tables.
Unfavorable outcome	A participant’s outcome will be classified as unfavorable if: • They are discontinued from their allocated study treatment and subsequently restarted on a different MDR-TB regimen; • Treatment is extended beyond the scheduled end of treatment for any reason other than making up of days when no treatment was given (missed treatment) for a maximum of 8 weeks. A maximum of 14 days of extra treatment (irrespective of reason) is acceptable before it is classified as treatment extension. In addition, if the phase of treatment has been extended for delayed sputum conversion (maximum 8-week extension permitted) the scheduled end of treatment will also be extended by the same amount; • They are restarted on any MDR-TB treatment after the scheduled end of treatment, but before 84 weeks after randomization; • More than 1 drug has been changed for any reason; • They die at any point during treatment or follow-up; • At least one of their last two culture results, from specimens taken on separate occasions, is positive;

The results of this trial for efficacy outcomes will be analyzed based on both modified intention-to-treat (*mITT*) and per protocol (*PP*) approaches with a primary consideration for *mITT* results. For the primary outcome analysis, the upper bound of the 95% confidence interval of the difference in proportion of favorable outcome between the WHO shorter regimen and the refined ultra-short regimen must be less than 10% (the margin of non-inferiority) in both the *mITT* and the *PP* populations for the ultra-short regimen to be declared non-inferior to the WHO shorter regimen group.

The analysis of sputum smear conversion will base on the conversion time after treatment initiation and the median time will be estimated in each group using the Kaplan-Meier method, and the difference in the distribution of time to culture conversion of the two regimens will be compared in a Cox proportional-hazard model. All AEs, serious AEs (SAEs) and treatment interruption due to adverse events will be collected and documented, and the proportion per type of AEs in the two regimens will be compared using the chi-square test or Fisher’s exact test.

### Confidentiality

Participants’ personal information will be limited to only information necessary for outcome evaluation of study regimen with laws on privacy protection and guaranteeing of confidentiality. Paper documents containing participants’ data will be locked in a specialized office in cooperative hospitals. Digital documents will be stored in password-protected files on website. Access to study documents will be restricted to authorized personnel only.

## Discussion

China accounted for 14% of the global total MDR/RR-TB in 2019, while treatment of RR/MDR-TB success rate only reached to 57% globally [1]. To improve the treatment outcome of RR/MDR-TB and optimize the regimens are urgently needed to work together worldwide.

Exploration of initiatives for MDR-TB started decades ago, but only increased in size and scope in the past few years. In 2020, WHO consolidated guidelines on TB (Module 4) revised current policy recommendations on treatment strategy for drug-resistant TB (DR-TB) [20]. The 2020 recommendations has witnessed two important trends in the MDR-TB treatment. One is the shorter duration of 6–12 months, founded on the Bangladesh regimen and the STREAM study. Another is the all-oral regimen in the strength of the effective novel and repurposed agents, following the promising results of the Nix-TB study. As a new recommendation, the shorter regimen where the injectable agent is replaced by bedaquiline is highly preferred for the patients with resistance to fluoroquinolones ruled out. However, the revised all-oral bedaquiline-containing regimen still includes seven agents, most of which are Group C agents. One pitfall of the regimen is the underappreciated role of the drug susceptibility testing (DST) for other agents except fluoroquinolones.

At present, a number of studies of shorter regimen for MDR-TB including MDR-END [21], endTB [22] and so on are carried out. The trends of MDR-TB treatment are short-term and full oral. However, how to optimize the patient’s treatment process and outcome based on DST has not been emphasized. Introducing rapid DST and more effective oral drugs to ensure drugs’ effect is a potential way to shorten and optimize the treatment. Especially DST of pyrazinamide, which plays a key role in reducing TB relapse rates and shortening the course of the disease treatment from 9 to 12 months to 6 months due to its powerful sterilizing activity [23, 24], is valuable and vital in those published studies [9, 25].

Our early study [9] provides excellent treatment success rate of precise regimen based on DST. It was the first prospective study that supported the superiority of molecular DST in guiding treatment decisions and refining treatment regimens. But the study was not designed in randomization, which means opinion was divided as to the impact of potential biases and whether these results could be replicated in different settings remains considering. TB-TRUST study is set up as a high- level evidence RCT study to evaluate the treatment outcome of precision treatment non-inferior to WHO shorter regimen, which uses rapid molecular testing to identify patients who can take shorter regimen with fluoroquinolone and shortens the treatment to 6 months for pyrazinamide sensitive patients based on the WGS result without introducing any new anti-TB medicines.

The 2018 WHO guidelines for DR-TB [7] proposed a three-drug combination treatment plan based on moxifloxacin, linezolid, and bedaquiline, which represented the arrival of the era of totally oral treatment. Nix-TB study with three oral drugs in 26 weeks achieved the favorable outcome in 90% patients, which means ensured effective drugs can achieve a 6-month shorter treatment regimen without too many drug, even for highly MDR-TB. However, not only novel developed medicines, but also traditional effective medicines with the susceptibility confirmed by DST are the key factors to ensure the effect of anti-MDR-TB drugs. Furthermore, a DST-guided precision treatment with traditional agents optimizes therapy outcome in more patients who cannot afford the expensive new medicines. With the rational use of anti-tuberculosis drugs, precision treatment can minimize and even avoid resistance amplification.

TB-TRUST study has few limitations. As a full-oral shorter regimen, without introducing bedaquiline is regrettably. Bedaquiline-containing regimens have achieved high conversion and success rates of MDR- and XDR-TB trials in published studies [8, 26, 27] and it is a popular and important agent in recent shorter regimen trials [22]. However, most Chinese patients cannot afford bedaquiline due to the high price and different medical insurance reimbursement ratio, which has affected its promotion in China. TB-TRUST study hopes to prove that accessible medicines are also effective with the guidance of DST and we believe that a bedaquiline-containing regimen under the precise DST will achieve better efficacy. Moreover, WGS based on TB cultured isolations is an accurate and effective method for DST of pyrazinamide at present. However, long period of acquiring the cultured isolations limits the acquisition of DST in time. To guide the design of regimens immediately and precisely in the beginning of therapy, it’s expectant that WGS or other innovative technology can be performed directly on clinical specimens without cultured isolations [28].

## Supplementary Information

**Additional file 1.**

## Data Availability

This is a study protocol manuscript, Not applicable.

## Abbreviations

AEs: Adverse events
AST: Aspartate transaminase
ALT: Alanine aminotransferase
BPNS: Brief peripheral neuropathy rating scale
CCO: Central coordinating office
DR-TB: Drug-resistant tuberculosis
DST: Drug susceptibility testing
MDR-TB: Multidrug-resistant tuberculosis
*mITT*: Modified intention-to-treat
MTB: *Mycobacterium tuberculosis*
PP: Per protocol
RCT: Randomized controlled Trial
RR-TB: Randomized controlled trial
SAEs: Serious adverse events
SLIDs: Second-line injectable drugs
WGS: Whole-genome sequencing
XDR: Extensively drug-resistant

## References

[1] WHO (2020). Global tuberculosis report 2020.

[2] Cox H, Hughes J, Black J, Nicol MP (2018). Precision medicine for drug-resistant tuberculosis in high-burden countries: is individualised treatment desirable and feasible?. Lancet Infect Dis.

[3] Van Deun A, Maug AKJ, Salim MAH, Das PK, Sarker MR, Daru P (2010). Short, highly effective, and inexpensive standardized treatment of multidrug-resistant tuberculosis. Am J Respir Crit Care Med.

[4] Kuaban C, Noeske J, Rieder HL, Aït-Khaled N, Abena Foe JL, Trébucq A (2015). High effectiveness of a 12-month regimen for MDR-TB patients in Cameroon. Int J Tuberc Lung Dis Off J Int Union Tuberc Lung Dis.

[5] Piubello A, Harouna SH, Souleymane MB, Boukary I, Morou S, Daouda M (2014). High cure rate with standardised short-course multidrug-resistant tuberculosis treatment in Niger: no relapses. Int J Tuberc Lung Dis Off J Int Union Tuberc Lung Dis..

[6] Nunn AJ, Phillips PPJ, Meredith SK, Chiang C-Y, Conradie F, Dalai D (2019). A trial of a shorter regimen for rifampin-resistant tuberculosis. N Engl J Med.

[7] Rapid Communication (2018). Key changes to treatment of multidrug- and rifampicin-resistant tuberculosis (MDR/RR-TB).

[8] Conradie F, Diacon AH, Ngubane N, Howell P, Everitt D, Crook AM (2020). Treatment of Highly Drug-Resistant Pulmonary Tuberculosis. N Engl J Med.

[9] Sun F, Li Y, Chen Y, Guan W, Jiang X, Wang X (2019). Introducing molecular testing of pyrazinamide susceptibility improves multidrug-resistant tuberculosis treatment outcomes: a prospective cohort study. Eur Respir J.

[10] New Rapid Molecular Test For Tuberculosis Can Simultaneously Detect Resistance To First- And Second-Line Drugs - Jul 16, 2020. Available from: http://cepheid.mediaroom.com/2020-07-16-New-Rapid-Molecular-Test-For-Tuberculosis-Can-Simultaneously-Detect-Resistance-To-First-And-Second-Line-Drugs. Accessed 11 Nov 2020.

[11] Yadon AN, Maharaj K, Adamson JH, Lai Y-P, Sacchettini JC, Ioerger TR (2017). A comprehensive characterization of PncA polymorphisms that confer resistance to pyrazinamide. Nat Commun.

[12] Liu W, Chen J, Shen Y, Jin J, Wu J, Sun F (2018). Phenotypic and genotypic characterization of pyrazinamide resistance among multidrug-resistant *Mycobacterium tuberculosis* clinical isolates in Hangzhou, China. Clin Microbiol Infect.

[13] Chen X, He G, Wang S, Lin S, Chen J, Zhang W (2019). Evaluation of whole-genome sequence method to diagnose resistance of 13 anti-tuberculosis drugs and characterize resistance genes in clinical multi-drug resistance mycobacterium tuberculosis isolates from China. Front Microbiol.

[14] Shi W, Chen J, Feng J, Cui P, Zhang S, Weng X (2014). Aspartate decarboxylase (PanD) as a new target of pyrazinamide in *Mycobacterium tuberculosis*. Emerg Microbes Infect..

[15] Zhang S, Chen J, Cui P, Shi W, Zhang W, Zhang Y (2015). Identification of novel mutations associated with clofazimine resistance in *Mycobacterium tuberculosis* : table 1. J Antimicrob Chemother.

[16] Zhang S, Chen J, Shi W, Liu W, Zhang W, Zhang Y (2013). Mutations in *panD* encoding aspartate decarboxylase are associated with pyrazinamide resistance in *Mycobacterium tuberculosis*. Emerg Microbes Infect.

[17] Zhang S, Chen J, Cui P, Shi W, Shi X, Niu H (2016). Mycobacterium tuberculosis mutations associated with reduced susceptibility to linezolid. Antimicrob Agents Chemother.

[18] Zhang Y, Zhang J, Cui P, Zhang Y, Zhang W (2017). Identification of novel efflux proteins Rv0191, Rv3756c, Rv3008, and Rv1667c involved in pyrazinamide resistance in mycobacterium tuberculosis. Antimicrob Agents Chemother.

[19] Zhang S, Chen J, Shi W, Cui P, Zhang J, Cho S (2017). Mutation in *clpC1* encoding an ATP-dependent ATPase involved in protein degradation is associated with pyrazinamide resistance in *Mycobacterium tuberculosis*. Emerg Microbes Infect..

[20] WHO (2020). WHO Consolidated Guidelines on Tuberculosis, Module 4: Treatment - Drug-Resistant Tuberculosis Treatment.

[21] Lee M, Mok J, Kim DK, Shim TS, Koh W-J, Jeon D (2019). Delamanid, linezolid, levofloxacin, and pyrazinamide for the treatment of patients with fluoroquinolone-sensitive multidrug-resistant tuberculosis (treatment shortening of MDR-TB using existing and new drugs, MDR-END): study protocol for a phase II/III, multicenter, randomized, open-label clinical trial. Trials..

[22] Khan U, Huerga H, Khan AJ, Mitnick CD, Hewison C, Varaine F (2019). The endTB observational study protocol: treatment of MDR-TB with bedaquiline or delamanid containing regimens. BMC Infect Dis.

[23] Gopal P, Grüber G, Dartois V, Dick T (2019). Pharmacological and molecular mechanisms behind the sterilizing activity of pyrazinamide. Trends Pharmacol Sci.

[24] Li S-Y, Tasneen R, Tyagi S, Soni H, Converse PJ, Mdluli K (2017). Bactericidal and sterilizing activity of a novel regimen with Bedaquiline, Pretomanid, Moxifloxacin, and pyrazinamide in a murine model of tuberculosis. Antimicrob Agents Chemother.

[25] Njire M, Tan Y, Mugweru J, Wang C, Guo J, Yew W (2016). Pyrazinamide resistance in mycobacterium tuberculosis: review and update. Adv Med Sci.

[26] Pym AS, Diacon AH, Tang S-J, Conradie F, Danilovits M, Chuchottaworn C (2016). Bedaquiline in the treatment of multidrug- and extensively drug-resistant tuberculosis. Eur Respir J.

[27] Borisov SE, Dheda K, Enwerem M, Romero Leyet R, D’Ambrosio L, Centis R (2017). Effectiveness and safety of bedaquiline-containing regimens in the treatment of MDR- and XDR-TB: a multicentre study. Eur Respir J.

[28] He G, Li Y, Chen X, Chen J, Zhang W (2020). Prediction of treatment outcomes for multidrug-resistant tuberculosis by whole-genome sequencing. Int J Infect Dis.

