# Supplementary material for: Refining MDR-TB treatment regimens for ultra short therapy (TB-TRUST): study protocol for a randomized controlled trial
Source: BMC Infect Dis. 2021 Feb 17;21:183. doi: 10.1186/s12879-021-05870-w (PMC7888137; doi:10.1186/s12879-021-05870-w)
Supplement: Supplementary file 1 — Additional file 1. [file 12879_2021_5870_MOESM1_ESM.docx]

# Additional file 1: Detailed schedule of patient monitoring exams and laboratory tests

|  | Baseline Visit | Week 2 | | Week 4 | Week 6 | Week 8 | | Week 12 | Week 16 | | Week 20 | | Until end of treatment | | | End of treatment | Post-treatment until 84 weeks after treatment initiation |
| --- | --- | --- | --- | --- | --- | --- | --- | --- | --- | --- | --- | --- | --- | --- | --- | --- | --- |
| Vital signs | X | X | | X | X | X | | X | X | | X | | Every 4 weeks | | | X | Every 12 weeks |
| physical examination | X |  | | X |  | X | | X | X | | X | | Every 4 weeks | | | X | Every 12 weeks |
| Brief peripheral neuropathy screen^1^ | X |  | | X |  | X | | X | X | | X | | Every 4 weeks | | | X | Every 12 weeks |
| Audiometry | X | Based on clinical requests | | | | | | | | | | | | | | |  |
| Visual acuity and color vision screen | X |  | | X | X | X | | X | X | | X | | Every 4 weeks | | | X | Every 12 weeks |
| Clinical assessment | X | X | | X | X | X | | X | X | | X | | Every 4 weeks | | | X | Every 12 weeks |
| Weight | X |  | | X | X | X | | X | X | | X | | Every 4 weeks | | | X | Every 12 weeks |
| Smear | X |  | | X | X | X | | X | X | | X | | Every 4 weeks | | | X | Every 12 weeks |
| Culture^2^ | X |  | | X | X | X | | X | X | | X | | Every 4 weeks | | | X | Every 12 weeks |
| Electrocardiogram | X | X | | X | X | X | | X | X | | X | | Every 4 weeks | | | X |  |
| Blood routine, AST, ALT | X | X | | X | X | X | | X | X | | X | | Every 4 weeks | | | X |  |
| Creatinine, uric acid, serum potassium | X | X | | X | X | X | | X | X | | X | | Every 4 weeks | | | X |  |
| Urine routine | X | X | | X |  | X | | X | X | |  | |  | | |  |  |
| TSH | X | Based on clinical requests | | | | | | | | | | | | | | |  |
| Blood sugar | X | Based on clinical requests | | | | | | | | | | | | | | |  |
| Pregnancy test | X | Based on clinical requests | | | | | | | | | | | | | | |  |
| HIV serostatus | X |  | |  |  |  | |  |  | |  | |  | | |  |  |
| Scales for anxiety and depression^3^ | X |  | | X |  | X | | X | X | | X | |  | | | X |  |
| Chest X-Ray | X | Based on clinical requests | | | | | | | | | | | | | | |  |
| DST by Xpert^®^ MTB/XDR | X |  |  | |  | |  |  | |  | |  | |  |  | |  |

1: Use a brief peripheral neuropathy screening scale (BPNS).

2: If the strain is positive after sputum culture, the traditional drug sensitivity test will be performed simultaneously.

3: Self-rating depression scale (SDS) for depression test, Self-rating Anxiety Scale (SAS) for anxiety test.
